# Community health promotion and medical provision and impact on neonates (CHAMPION2) and support to rural India’s public education system and impact on numeracy and literacy scores (STRIPES2): an update to the study protocol (v 11) for a cluster randomised trial in Madhya Pradesh, India

**DOI:** 10.1186/s13063-024-08245-z

**Published:** 2024-07-08

**Authors:** Siddharudha Shivalli, Ila Fazzio, Chris Frost, Diana Elbourne, Nicholas Magill, Dropti Sharma, Sajjan Singh Shekhawat, Rukmini Banerji, Sridevi Karnati, Harshavardhan Reddy, Padmanabh Reddy, Rakhi Nair, Madan Gopal, Peter Boone

**Affiliations:** 1 London School of Hygiene and Tropical Medicine, London, UK; 2 Effective Intervention, London, UK; 3 Pratham Education Foundation, New Delhi, India; 4 GH Training and Consulting, Hyderabad, India; 5 NICE Foundation, Hyderabad, India

**Keywords:** Cluster randomised controlled trial, India, Neonatal mortality, Immediate neonatal care, Postnatal care, Maternal mortality, Stillbirths, Primary education, Supplementary teaching, Literacy, Numeracy

## Abstract

**Background:**

This update outlines amendments to the CHAMPION2/STRIPES2 cluster randomised trial protocol primarily made due to the COVID-19 pandemic and nationwide lockdown in India in 2020. These amendments were in line with national guidelines for health research during the COVID-19 pandemic.

**Methods:**

We did not change the original trial design, eligibility, and outcomes. Amendments were introduced to minimise the risk of COVID-19 transmission and ensure safety and wellbeing of trial staff, participants, and other villagers. CHAMPION2 intervention: participatory learning and action (PLA) and fixed day service (FDS) meeting were revised to incorporate social distancing and hygiene precautions. During the COVID-19 pandemic, PLA participation was limited to pregnant women and birthing partners. STRIPES2 intervention: before/after-school classes were halted for a period and then modified temporarily (reducing class sizes, and/or changing meeting places) with hygiene and safe distancing practices introduced. Data collection: The research team gathered as much information as possible from participants by telephone. If the participant had no telephone or could not be contacted by telephone, data were collected in person. COVID-19 precautions: trial teams were trained on COVID-19 precautions and used personal protective equipment whilst in the villages for trial-related activities.

After restarting the trial between June and September 2020 in a phased manner, some trial activities were suspended again in all the trial villages from April to June 2021 due to the second wave of COVID-19 cases and lockdown imposed in Satna, Madhya Pradesh. Trial timelines were also revised, with outcomes measured later than originally planned.

**Trial registration:**

Clinical Trial Registry of India CTRI/2019/05/019296. Registered 23 May 2019.

https://ctri.nic.in/Clinicaltrials/pmaindet2.php?EncHid=MzExOTg=&Enc=&userName=champion2.

## Introduction

CHAMPION2/STRIPES2 was a cluster randomised controlled trial with villages (clusters) receiving either a health (CHAMPION2) or education (STRIPES2) intervention. The CHAMPION2 control villages received the usual health services (plus the STRIPES2 intervention) and the STRIPES2 control villages received the usual education services (plus the CHAMPION2 intervention). All the trial activities were suspended on 25 March 2020 due to a nationwide lockdown in India considering the COVID-19 pandemic. The trial activities were restarted with modifications in a phased manner from June to September 2020. This update outlines the amendments to the previously published CHAMPION2/STRIPES2 trial protocol [1], which were introduced to minimise the risk of COVID-19 transmission and to attempt to ensure the safety and wellbeing of trial staff, participants, and other villagers. These amendments included modifying the intervention components and data collection methods without any changes in the original cluster trial design, eligibility criteria, and outcome measures. These amendments were designed by the trial management group (independent implementation, data collection, and research teams), were in line with national guidelines for health research during the COVID-19 pandemic [2], and were approved by the ethics and trial steering committees (TSC). These amendments were implemented by the same implementation and data collection teams as mentioned in the previously published CHAMPION2/STRIPES2 trial protocol (i.e. CHAMPION2 intervention: NICE foundation; STRIPES2 intervention: Pratham Education Foundation; data collection: GH Training and Consulting, GHTC) [1].

## Methods

### Eligibility criteria

#### Villages (clusters)

One of the eligibility criteria for a village was that its centre should not be within a 5-km radius of a Community Health Centre (CHC) or Civil Hospital (CH), as such villages are already well served by the local health services. At the time of the initial identification of eligible villages, we did not take account of the location of one CH (at Maihar). We subsequently discovered that one of our selected villages was less than 5 km (but more than 4 km) from this CH. Since the village had already been informed about the trial, we decided that the village should be retained and randomised.

#### CHAMPION2

There was no change in the eligibility criteria for women. For the trial analysis, we had initially planned to start counting births and deaths 12 months after randomisation (i.e. from 19 June 2020). This lag in measurement was necessary to ensure adequate exposure to the intervention considering both the time interval between conception and birth, and the fact that training and establishment of services in intervention villages would take around 6 months. However, due to the COVID-19 pandemic interruptions to the trial activities, we decided to start counting births and deaths on 1 January 2021 (approximately 18 months after randomisation).

#### STRIPES2

There was no change in the eligibility criteria for children. In the previous protocol version, we used the term ‘parent’ to describe the person primarily responsible for a child’s care. For clarity, we have updated this term to ‘caregiver’ (i.e. parent or a person primarily responsible for the child).

### Additional consent provisions for collection and use of participant data

With the additional measures taken following the trial restart in July 2020 to minimise the risk of COVID-19 transmission, household heads and enrolled men and women were asked for permission to, when feasible, conduct interviews by telephone instead of face to face.

During the first telephone contact with all participants, a tele-enumerator (TE) sought the participant’s consent to gather information over the phone, and this consent was recorded. We did not record any other phone calls with participants.

### Intervention amendments

#### CHAMPION2 (health) intervention

With the re-start of the trial in June/July 2020, the NICE Foundation modified the CHAMPION2 intervention to reduce the risk of COVID-19 transmission. The participatory learning for action (PLA) and fixed day service (FDS) meeting plans were revised to incorporate government recommended social distancing and hygiene procedures. The pregnant women attending FDS were staggered to minimise the waiting period and crowding with safe distancing and hygiene practices adopted. Participant discussion groups (PDGs) were conducted one-to-one with pregnant women. PLA meetings of participants were limited to pregnant women and their birthing partners. A component was added in the PLA to teach participants about means of reducing the risk of COVID-19 transmission. Staggering of FDS and limiting the participants for PLA were removed once COVID-19 restrictions were lifted in Satna from August 2021.

The following new activities were conducted to improve the effectiveness of PDGs and PLA participation: From March 2022, after each PDG session, nurses started distributing a small sheet of paper to emphasise key messages or actions, helping mothers to remember what they had learned in that session. From October 2022, public announcements were started in the villages to improve the PLA attendance, to connect to more birthing partners and healthcare in pregnancy decision makers, to bring in more awareness about the need for institutional deliveries, and to improve the overall health seeking behaviour in the community.

Due to the COVID-19 pandemic interruptions, the timeline in which CHAMPION2 outcomes counted was changed (details in the ‘Outcomes’ section).

#### STRIPES2 (education) intervention

This was restarted from August 2020 in a phased manner by the Pratham Education Foundation with the following modifications.

##### Remote contacting

From August 2020, Pratham instructors (PIs) started sharing short messages to caregivers’ phones daily to help children learn and have fun during difficult times. The messages were short, interactive, and engaging and served as a stepping stone for building interest. The messages were initially simple, and over time became more complex. The content of messages was developed considering the literacy, numeracy, and other areas of holistic development (e.g. social, emotional, physical, and creative) of children and was relevant to their everyday lives. Besides sharing messages, instructors followed up with caregivers by telephone daily, focusing on how they and their children were keeping during these circumstances (including but not limited to how caregivers could help their children study whilst at home).

##### Small group classes

Abiding by the COVID-19 guidelines, between November 2020 and April 2021, PIs started conducting small-group classes. These small group classes included 4–5 children and were conducted for about 20–30 min. During classes, PIs also discussed the risks of COVID-19 and means of reducing transmission with their students. Also, caregivers were asked not to send children to the classes if their child or anyone in their household had symptoms suggestive of COVID-19. Along with small group PI classes, separate individual and small group interactions with children, and their caregivers were done to engage caregivers in their children’s learning, understand learning levels of children after a prolonged break, and prepare children to resume small group classroom activities.

The mother engagement activities (mothers’ meetings and home visits) also had to be suspended due to COVID-19. In late November and early December 2020, PIs restarted the bi-weekly mothers’ meetings and home visits. In these meetings, PIs distributed learning materials (or mother’s kits, on subjects such as picture reading, pattern, comparison, etc.) and demonstrated to mothers how to do some activities with their children. Mothers would then practice in groups so they could do the activities at home with their children. PIs visited the house for follow-up after 2–3 days to learn about the previous activities and demonstrate new activities to mothers using the mother’s kits.

The STRIPES2 activities (except remote contacting and home visits) were suspended again in April 2021 due to the second wave of COVID-19 cases and lockdown imposed in Satna, Madhya Pradesh. Regular before/after-school classes (as conducted before COVID-19) were restarted in July 2021. Weekly before/after-school classes (of 20–30 min duration) were conducted for some children who could not join the daily PI classes due to various reasons such as the PI class being far away, parent not willing to send the child, etc.

Post COVID-19 (from July 2021), besides before/after-school classes, mothers’ meetings, and home visits, Pratham continued to send short messages to caregivers of all the children. PIs followed up on alternate days with only those children who could not attend the daily PI classes.

The following additional volunteer-led activities were started in early 2022 to ensure continued learning:

- Fun activity in the community: To strengthen children’s learning and community involvement in the education process, from January 2022, 163 trained young volunteers conducted the various fun activities, including storytelling. One day of face-to-face training was provided to volunteers on creating materials for storytelling, such as puppets, and incorporating elements like voice modulation and body gestures as an effective way to make learning enjoyable and interactive. The regularity and duration of the fun activities, conducted in small groups of 4–5 children, were intended to provide a focused and personalised approach. The volunteers were trained in ‘First Aid’ in exchange for their voluntary participation. This not only benefited the volunteers personally but also added value to the community by increasing overall preparedness for emergencies.
- Library at local shop: In March 2022, the community libraries at local shops were established to support and encourage the reading habits among the community members, particularly children. The goal was to provide a convenient platform for borrowing or issuing authentic children’s literature. In each intervention village, local shops played a vital role by serving as platforms where a diverse range of children’s literature to strengthen the reading, writing, and learning were stored. The variety of children’s literature available in these community libraries complemented various tasks aimed at developing basic foundational skills and fostering creativity among children. The setup allowed children to easily borrow these materials from shops within their proximity, creating a convenient and accessible resource for their educational and creative needs.
- Digital tablet-based activity: To reinforce learning with the help of tablets, tablet-based activities had been planned initially before COVID-19. However, they were delayed due to the COVID-19 pandemic and were started in April 2022. Sixty-five trained volunteers conducted mapped games in 65 villages daily for 25–30 min with groups of 4–5 children. Apart from the fun activities, other activities were meant to reinforce and support the learning of children struggling with decoding and number recognition skills through games. A new language and maths activity was done with groups of 4–5 children every day.
- STRIPES2 intervention internal assessments: The assessments were conducted throughout the trial period in the following months: December 2019, November 2020, April 2021, August 2021, February 2022, and June 2022. Because of COVID-19, a few additional assessments that had not been initially planned were conducted.

Due to the interruptions by the COVID-19 pandemic, the STRIPES2 intervention finished later than had initially been planned (details in the ‘Outcomes’ section).

### Outcomes

#### CHAMPION2

We did not change the outcomes. However, due to the COVID-19 pandemic interruptions, the timeline in which these outcomes counted was changed. Initially it was 30 months from 19 June 2020, this being changed to 30 months from 1 January 2021, meaning that different pregnant women (particularly new women entering the trial by marrying enumerated unmarried men) contributed to the CHAMPION2 analyses than would otherwise have been the case. Further, due to the COVID-19 pandemic, the independent data collection team (GHTC) gathered information from participants as much as possible by telephone. If the participant did not have a telephone or could not be contacted by telephone, data were collected in person by visiting the household.

Mortality outcomes, serious adverse events (SAE) (hospitalisation and maternal blood transfusion) during pregnancy and in the 24 h after delivery continued to be collected for all pregnancies that reached or passed 28 weeks of gestation. After this period, hospitalisation and blood transfusion data were collected in all cases of maternal or neonatal deaths. However, to minimise the risk of COVID-19 transmission during household visits, we collected all other secondary outcomes for only a subsample of births. The subsample comprised (i) all births from pregnancies that went beyond 28 weeks of gestation and where one or more babies died (stillbirth or neonatal death), (ii) all multiple births from pregnancies that went beyond 28 weeks of gestation and (iii) 10% of women whose pregnancies went beyond 28 weeks of gestation and where the baby survived the neonatal period. The 10% was selected by randomising women whose babies survived the neonatal period in a 1:9 ratio. Women were not re-randomised, so if selected to be asked to give data on all secondary outcomes for their first singleton baby who survived the neonatal period, they were also selected to give details on all secondary outcomes for all subsequent births etc. The subsample selection computer programme was designed by the database team in the UK. The selection was stratified by village and used a block size of 10. The item response options in questions related to antenatal and postnatal care were also revised to allow investigation of the extent to which the pandemic had an impact on the uptake of health services.

### STRIPES2

We did not change the outcomes. However, due to the COVID-19 pandemic interruptions, the STRIPES2 intervention finished later than had initially been planned, meaning that the intervention (and the final testing from July to September 2022) was carried out in older children than would otherwise have been the case.

### Participant timeline

#### Duration

##### CHAMPION2

The CHAMPION2 intervention had been planned for 3 ½ years initially; however, it was implemented for an additional 6 months (4 years in total) as the timeline in which the outcomes counted was changed, i.e. initially it was 30 months from 19 June 2020 which was changed to 30 months from 1 January 2021.

##### STRIPES2

Initially, we had planned to implement the STRIPES2 intervention for 17 months. However, owing to the interruptions by the COVID-19 pandemic, the STRIPES2 intervention was extended by a little over a year with the endline test carried out between 24 July and 19 September 2022 rather than April–May 2021 as had been initially planned. In the STRIPES2 trial, there were three distinct phases as described below:

##### Pre-COVID-19 (October 2019–March 2020)

In October 2019, PIs started the warm-up phase with various planned activities and started the daily PI classes in November 2019. All the trial activities were suspended from 20 March 2020 due the COVID-19 lockdown.

##### During COVID-19 (April 2020-June 2021)

No activities were conducted from mid-March–July 2020. From August 2020, remote contacting started. From November 2020, small group classes were started. The mother engagement activities (mothers’ meetings and home visits) were started in late November and early December 2020. The STRIPES2 activities (except remote contacting) were suspended again from April to June 2021 due to the second wave of COVID-19 cases and lockdown in Satna.

##### Post COVID-19 (July 2021–June 2022)

Before/after-school PI classes, mothers’ meetings, and home visits restarted. Weekly PI classes were conducted for those children who could not join the daily PI classes. Remote contacting continued for those children who could not join PI classes. Digital tablet-based activities and new volunteer-led activities (fun in the community and library at local shop) were started in April, January, and March 2022, respectively.

STRIPES2 midline testing of children (December 2021–January 2022) and a caregiver survey (February–March 2022) were conducted later than had initially been planned due to the COVID-19 pandemic.

An updated timeline with the key events during the trial is presented in Table 1.

**Table 1:** Updated timeline of the key events in the CHAMPION2/STRIPES2 trial

Date	Event
December 2015	Protocol submitted to the L V PRASAD EYE Institute (LVPEI) and London School of Hygiene and Tropical Medicine (LSHTM) Ethics Committees
January to October 2016	Permits sought and obtained from the Departments of Health and Education in Madhya Pradesh (Mission Director from the National Health Mission, Director of the Child Health for Madhya Pradesh, Additional Mission and Director of the Education Department)
February 2017	Resubmission of protocol and short report to LVPEI and LSHTM Ethics Committees and Madhya Pradesh government
March to May 2017	Village consent, village mapping and piloting of enumeration forms
July 2017 to January 2018	Enumeration of the participants and data entry
September 2018 to April 2019	Health Ministry’s Screening Committee (HMSC)/ Indian Council of Medical Research (ICMR) application and approval
November 2018 to January 2019	Knowledge, Attitude and Practice (KAP) survey interviews and data entry
April to June 2019	Catch-up enumeration
19 June 2019	Randomisation
June to December 2019	Intervention design, village sensitisation and training
October 2019	STRIPES2 intervention started
December 2019	CHAMPION2 intervention started
March 2020	Trial was suspended due to COVID-19
June 2020	Trial protocol and document reporting changes due to COVID-19 submitted to LVPEI and LSHTM Ethics Committees, Madhya Pradesh government and ICMR
July 2020	CHAMPION2 activities adapted to COVID-19 and restarted
August 2020	STRIPES2 activities adapted to COVID-19 and restarted
1 January 2021	CHAMPION2 neonatal and maternal survival start to count to final analysis
December 2021–January 2022	STRIPES2 midline tests for children
February–March 2022	STRIPES2 midline caregiver survey
30 June 2022	STRIPES2 intervention ended
24 July 2022–19 September 2022	STRIPES2 endline testing (EGRA and EGMA)
November–December 2022	STRIPES2 endline caregiver survey
June–July 2023	Informing trial participants and village heads about trial winding down
30 June 2023	CHAMPION2-stop counting neonatal and maternal survival
1 July 2023	CHAMPION2-stop enrolling new pregnant women. Only FDS for enrolled pregnant women in the intervention villages
October 2023	STRIPES2 statistical analysis completed
July–October 2023	CHAMPION2-final data collection of neonatal and maternal survival for those whose pregnancy ended on/before 30 June 2023
31 December 2023	Stop fixed day services in the CHAMPION2 intervention villages
July 2024	CHAMPION2-statistical analysis completed

### Sample size

There were no revisions to the sample size calculations since data collection for the primary and key secondary outcomes were not materially altered. Minor corrections to wording were made. The corrected paragraph is as follows.

The process by which clusters (villages) were selected is described under ‘Recruitment’. After application of the first three criteria to identify clusters (steps 1 to 3), there were 484 villages that were potentially eligible for the trial. Originally, it had been the intention to randomise 300 villages, because this gave over 90% statistical power to detect (1) a 20% reduction in neonatal mortality in CHAMPION2 and (2) a difference of 0.25 SD in mean standardised test scores in STRIPES2. However, incorporating the buffer zones meant that only 204 villages could be selected. These 204 villages had a mean population of 1487 (minimum 558, maximum 2490) and SD of 505 (equating to a coefficient of variation of 0.34). Estimating the number of children in each school year from the number younger than 6 years (divided by 6), the mean number of children in each school year was 38.3 (minimum 20, maximum 71) with SD of 13.3 (a coefficient of variation of 0.35). Assuming that 25% of the children would not be eligible according to the criteria, this gave an estimated mean number of eligible children per village of 28.7 with a minimum of 15.

In CHAMPION, the design effect for neonatal mortality was 1.306, equating to an intra-cluster correlation coefficient (ICC) of 0.011 (with allowance for variability in cluster size, the assumed coefficient of variation = 0.34). For CHAMPION2, allowing for the fact that each village had an average population of 1487 and estimated crude birth rate of 30.7 per 1000 population per year in rural areas of Satna district [3], 114 births per village over a 30-month follow-up period were expected. Assuming (1) an ICC of 0.011 for the primary outcome, (2) an assumed coefficient of variation for village size variability of 0.34, (3) that 5% of villages would be excluded for reasons such as withholding consent and (4) that there would be 10% loss to follow-up, a trial with 194 villages (95% of 204) had 75% power (5% 2-sided significance) to detect a 20% reduction in neonatal mortality from 6.7% to 5.36 and 91% power (5% two-sided significance) to detect a 25% reduction in neonatal mortality from 6.7 to 5.0% (this and other power and sample size calculations were performed using the ‘clustersampsi’ command in Stata 14). Since the reduction in neonatal mortality seen in CHAMPION was 25%, proceeding with 204 villages seemed reasonable given the requirement for buffer zones in order to avoid contamination.

We estimated that the 204 villages would include an average of 28.7 eligible students (5855 students in total). In the STRIPES trial, the estimated effect was a 0.75 SD increase in mean score: however, effects of smaller magnitude than this would still be important to detect. Conservatively assuming that 60% of the eligible children (17 per cluster, 3468 students in total) would take the test at the end of the trial and an ICC of 0.23 (as seen in the STRIPES trial) then a trial with 194 villages (i.e. assuming that 5% of the 204 villages would not take part) would give 88% power to detect a difference of 0.25 SD in mean standardised scores between intervention and control villages using a conventional two-sided significance level of 5% (assuming a coefficient of variation of 0.35 in numbers taking the test by village). If the treatment effect is of the order of that seen in the STRIPES trial then there will be reasonable statistical power to explore interactions by ethnicity, gender, wealth, and geographic location.

As described above, in the sample size calculation we anticipated that 194 of the 204 villages would be randomised. In fact, 196 were randomised with 6 villages being removed since they were found to be too close to urban areas to be considered rural, and 2 removed because insufficient eligible children were found.

### Statistical analysis

Following the advent of COVID-19, some secondary outcomes were collected for only a subsample of births. The subsample comprised (i) all births from pregnancies that went beyond 28 weeks of gestation and where one or more babies died (stillbirth or neonatal death), (ii) all multiple births from pregnancies that went beyond 28 weeks of gestation, and (iii) 10% of women whose pregnancies went beyond 28 weeks of gestation and where the baby survived the neonatal period (details of the selection process are given in the ‘Outcomes’ section). Summary statistics for such secondary outcomes will be appropriately weighted to take into account this sampling strategy.

### Data collection and management

Based on the revisions to the methodology during the COVID-19 pandemic, GHTC established a tele-calling centre and trained a team of tele-callers to collect monthly monitoring data. GHTC worked with the database team in the UK (Sealed Envelope) to syncronise the tele-calling system with the CHAMPION2 monitoring system. GHTC gathered as much information as possible from participants by telephone. If the participant did not have a telephone or could not be contacted by telephone, data were collected in person by visiting the household. Some survey instruments to collect data on the outcomes were modified, the language and terminology used were re-assessed to suit telephone-based interview.

The key details of changes in the data collection methods are as follows:

- Monthly monitoring of women and unmarried men: aim was to identify women who become pregnant and men who get married (to enrol his wife in the trial). Post amendment, a tele-enumerator (TE) collected the data by phone. When this was not possible, the village enumerator (VE), resident in the same village, interviewed women and unmarried men in person with all the COVID-19 precautions.
- Monthly monitoring of pregnant women: aim was to identify women whose pregnancy reached or passed 28 weeks of gestation. Post amendment, a TE collected the data by phone. When this was not possible, the VE interviewed women in person with all the COVID-19 precautions.
- Information on outcome of pregnancy with ≥ 28 weeks of gestation: aim was to capture information on liveborn/stillbirths, neonatal mortality, maternal mortality, and other secondary outcomes as described in the protocol [1]. Post-amendment, a TE collected the data on the phone regarding primary outcome and key secondary outcomes. Where this was not possible, the data supervisor (DS) from GHTC interviewed women in person taking all the COVID-19 precautions. For other secondary outcomes, the DS only interviewed the mother for a subsample of pregnancies as described above in the section on outcomes.

Whenever in-person interviews were conducted, the interviewer started by asking whether the interviewee had any symptoms suggestive of COVID-19. If he/she had symptoms suggestive of COVID-19, the interviewer completed the (very short) monitoring forms but postponed any longer outcome-related interviews for at least 14 days and advised the interviewee to seek medical care. The interviewer always wore a mask, maintained a safe distance, and practised good hygiene.

### Additional plans to reduce risk of COVID-19 transmission

#### COVID-19 precautions

All trial staff were trained to minimise COVID-19 transmission through good hygiene practices, social distancing, and wherever possible avoiding face-to-face meetings. This training was organised by the NICE Foundation (the partner leading the health intervention), through video calls and small group trainings. Trial staff used personal protective equipment (facial mask, gloves, and hand sanitiser) whilst in the villages for trial-related activity.

### Oversight and monitoring

#### COVID-19 monitoring

As per the TSC recommendation, we explored the collection of data on COVID-19 cases in villages through telephone calls, village enumerators, and ASHAs. However, it was not possible to collect reliable data on COVID-19 cases in villages due to practical issues like the lack of a uniform definition of a COVID-19 case, confidentiality, associated stigma, etc. Hence, we (including TSC) later agreed that all the three trial implementation partners in India would report to the trial coordinator (TC) if there were any COVID-19 cases reported (in any trial village or among the project staff) or if lockdown was imposed by the local government. The TC coordinated with trial staff to follow up any cases and investigated if they were related to trial activities.

## Trial status

STRIPES2: On 30 June 2022, the STRIPES2 intervention ended as planned [Table 1]. Endline testing (July–September 2022) and endline parental surveys (November–December 2022) were completed as planned. We completed the STRIPES2 analysis in October 2023.

CHAMPION2: We stopped enrolling new pregnant women into the trial in May 2023. On 30 June 2023, we stopped counting neonatal and maternal survival. Data on trial outcomes for the enrolled women, whose pregnancy ended on/before 30 June 2023, were collected from July to October 2023. The trial participants and village heads were informed about the winding down process. Only the FDS component of the CHAMPION2 intervention continued until 31 December 2023 for the enrolled women who reported a pregnancy before 1 July 2023. We now intend to complete the analysis by July 2024.

## Data Availability

Data sharing is not applicable to this article (a protocol update) as no datasets will be generated or analysed during this stage of the study. After publication of the initial results, the anonymised datasets used and/or analysed during the trial with relevant statistical code will be available from the corresponding author on reasonable request.

## Abbreviations

CH: Civil Hospital
CHC: Community Health Centre
COVID-19: Coronavirus disease
DS: Data supervisor
EI: Effective Intervention
FDS: Fixed Day Services
GHTC: GH Training and Consulting
ICMR: Indian Council of Medical Research
ICC: Intra-cluster correlation coefficient
KAP: Knowledge Attitudes Practice
LSHTM: London School of Hygiene and Tropical Medicine
LVPEI: L V PRASAD EYE Institute
NGO: Non-Governmental Organisation
PDG: Participatory Group Discussion
PI: Pratham Instructor
PLA: Participatory Learning and Action
TC: Trial coordinator
TSC: Trial steering committee
TE: Tele-enumerator
VE: Village enumerator

## References

[1] Agarwal A, Banerji R, Boone P, Elbourne D, Fazzio I, Frost C, Gopal M, Karnati S, Nair R, Reddy H, Reddy P, Sharma D, Shekhawat SS, Shivalli S (2020). Protocol for a cluster randomised trial in Madhya Pradesh, India: community health promotion and medical provision and impact on neonates (CHAMPION2); and support to rural India's public education system and impact on numeracy and literacy scores (STRIPES2). Trials.

[2] Mathur R, editor. National Guidelines for Ethics Committees Reviewing Biomedical and Health Research during COVID-19 Pandemic. Indian Council of Medical Research-National Centre for Disease Informatics & Research, Bengaluru; 2020 Apr. Available from: https://www.icmr.gov.in/pdf/covid/techdoc/EC_Guidance_COVID19_06052020.pdf. Cited 2023 May 18.

